# Exploring risk factors for totally implantable venous access devices (TIVADs)-related thrombotic occlusion in the off-treatment period

**DOI:** 10.1038/s41598-023-37902-7

**Published:** 2023-07-04

**Authors:** Lei Liu, Zhanlun Liu, Jianxin Wang, Meng Cheng, Yanli Xie, Weina Wang, Cuicui Ma, Lina Zhang, Zhenchuan Song, Yanshou Zhang

**Affiliations:** 1 Breast Center, The Fourth Hospital of Hebei Medical University, Shijiazhuang, 050011 Hebei Province China; 2 Hebei Province Hospital of Traditional Chinese Medicine, Shijiazhuang, Hebei Province China

**Keywords:** Breast cancer, Risk factors

## Abstract

Totally implantable venous access devices (TIVADs) have been widely used for many years in the management of patients suffering from cancer. Thrombotic occlusion is the most common functional complication in the off-treatment period. This study aims to investigate the incidence of and risk factors for TIVADs-related thrombotic occlusion in patients with breast cancer. The clinical data of 1586 eligible patients with breast cancer with TIVADs at the Fourth Affiliated Hospital of Hebei Medical University from 1 January 2019 to 31 August 2021 were analysed. Thrombotic occlusion was confirmed by angiography with signs of partial or total occlusion. Thrombotic occlusion occurred in 96 (6.1%) cases. Multivariable logistic regression analysis showed that the insertion site of the catheter (*P* = 0.004), size of the catheter (*P* < 0.001), and indwelling time (*P* < 0.001) were significant factors for thrombotic occlusion. Insertion in the right internal jugular vein, smaller catheter size and shorter indwelling time can lower the incidence of thrombotic occlusion in breast cancer patients with TIVADs in the off-treatment period.

## Introduction

Central venous access has gained wide acceptance for cancer patients due to its low infection rate, long indwelling time, and easy maintenance protocol. Among all types of central venous catheters, totally implantable venous access devices (TIVADs) have become routine in the management of patients suffering from breast cancer, as they facilitate the administration of chemotherapy agents, blood products, high-concentration medicine, and other cytotoxic agents, avoid irritating peripheral blood vessels and provide patients with a better cosmetic appearance^1–4^.

TIVADs are a permanent port-a-cath system in which a central venous catheter is connected to a reservoir implanted on the patient’s chest or arm through surgical methods^2^. After completing treatments, patients can choose removable TIVADs or long-term indwelling TIVADs with regular maintenance. A study has shown that breast cancer patients have a high risk of recurrence or metastasis within the first two years^3^. Therefore, removal of TIVADs is commonly postponed by patients after the end of curative chemotherapy or adjuvant chemotherapy for the purpose of reusing the device and reducing the financial cost of reinsertion due to disease recurrence. During the maintenance period, TIVADs are regularly flushed by nurses to keep the catheter patent. Aspirating blood return from the catheter can be an effective sign to judge the functional status of the catheter^2^.

The most common functional complication of TIVADs in the off-treatment period is occlusion^4,5^. Occlusion can arise from precipitation of medications or parenteral nutrition, mechanical obstruction, or thrombotic causes. The basis of thrombotic occlusion can be fibrin sheath formation due to accumulation of fibrin around the catheter tip or intraluminal thrombosis due to the accumulation of blood clots within the catheter^2^, which can occur separately or in combination^5^. Although the locations of thrombosis are different, they share similar symptoms, which include difficulty in aspirating blood with or without infusion difficulties^6^. Mechanical occlusion can be solved through case-specific therapy, while clearing the blood clots or fibres has become the first option for thrombotic occlusion^4^. However, this method is usually time-consuming for medical teams, especially for oncology nurses who are involved in the long-term follow-up of patients with TIVADs^5^. A study has shown that oncology nurses need to spend an extra 27.1 min to 29 min dealing with patients with thrombotic occlusion^7^. Moreover, many other methods, such as systematic anticoagulant therapy or utilization of heparin-boned catheters, have been reported to be largely ineffective. It is worth noting that some risk factors related to thrombotic occlusion are preventable^8^. Therefore, this study aimed to explore the risk factors for TIVAD-related thrombotic occlusion to initiate early interventions and prevent the occurrence of thrombotic occlusion.

## Results

### General characteristics of enrolled patients

**Figure 1:**
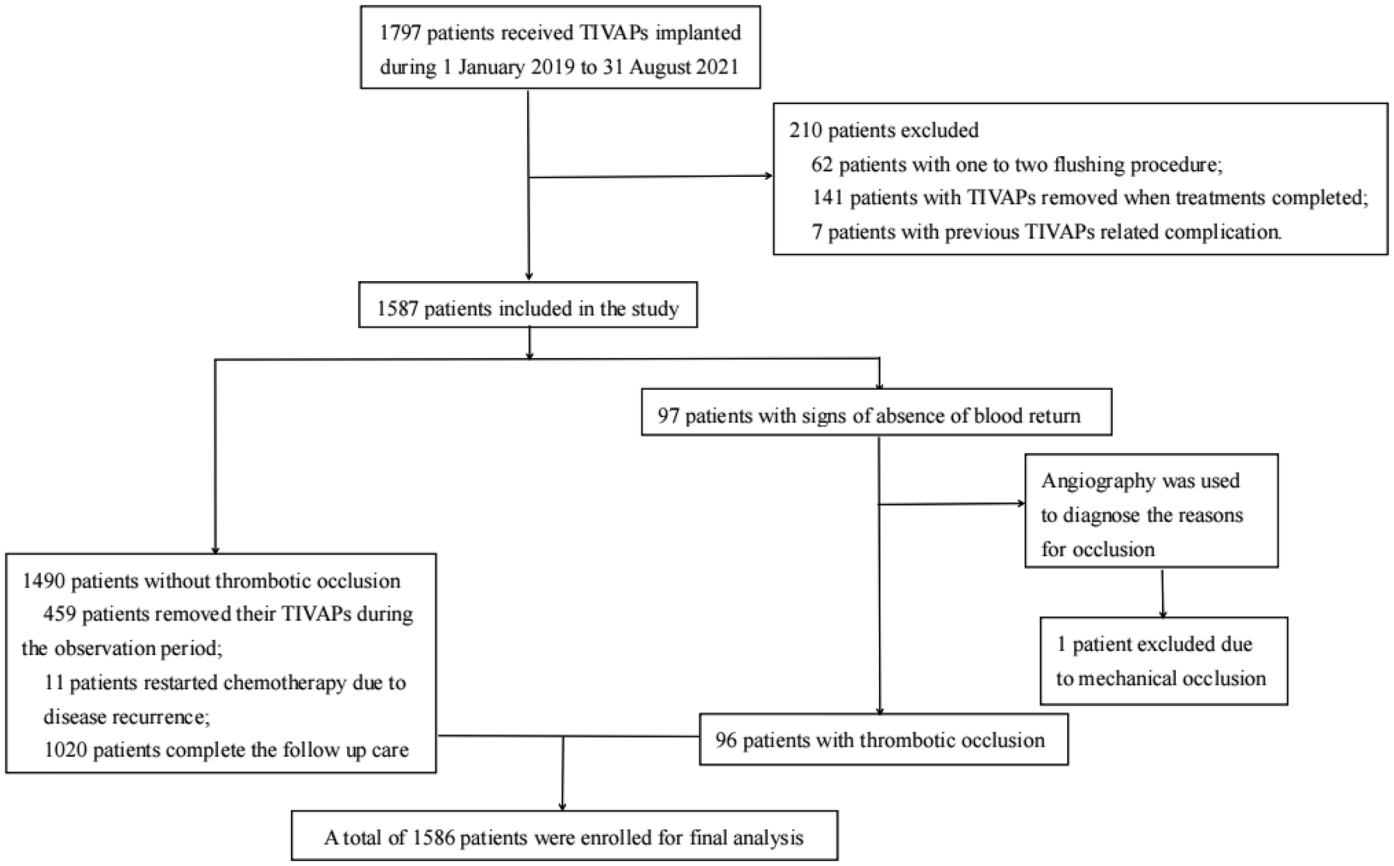
Selection process and characteristics of enrolled participants.

Of 1797 breast cancer patients, 62 patients had only one maintenance procedure, 141 patients underwent TIVAD removal when treatments were completed, 7 patients had previous TIVAD complications (3 pocket infections and 4 fibrin formations), and one had mechanical occlusion (the catheter tip migrated to the internal jugular vein). A total of 1586 patients were enrolled in our study (see Fig. 1 for the selection process and characteristics of the enrolled participants).

Of these cases, 96 patients were found to have thrombotic occlusion, with an incidence of 6.1% (96/1586). Among the 96 patients, 89 presented with withdrawal occlusion, which was defined as sluggish or absent blood return, but infusion of fluid was possible^1^ 7 patients presented with total occlusion, which was defined as inability of infusion and aspiration^1^. Compared to nonthrombotic occlusion patients, thrombotic occlusion patients presented significant differences in insertion site (*P* = 0.005), catheter size (*P* < 0.001), catheter materials (*P* < 0.001), and indwelling time (*P* = 0.010). Surprisingly, maintenance interval was not a risk factor for thrombotic occlusion during the analysis, and the incidence of thrombotic occlusion was 6.1% (31/506) for patients who underwent maintenance every one month, 7.8% (39/502) for every two months, 2.3% (3/129) for every three months, 2.9% (5/172) for every four months, and 6.5% (18/277) for irregular intervals. There was no significant difference among the different flushing groups (*P* = 0.065) (Table 1).

**Table 1:** Clinical features of breast cancer patients with and without TIVAD-related thrombotic occlusion.

Variables		Nonocclusion group (n = 1490)	Occlusion group (n = 96)	*P* value
Age at implantation, years	≤ 55	1098 (73.7%)	65 (67.7%)	0.199
	> 55	392 (26.3%)	31 (32.3%)	
BMI	< 25	695 (46.6%)	50 (52.1%)	0.585
	25–30	642 (43.1%)	37 (38.5%)	
	≥ 30	153 (10.3%)	9 (9.4%)	
Menopausal status, n (%)	Pre-menopausal	952 (63.9%)	63 (65.6%)	0.732
	Post-menopausal	538 (36.1%)	33 (34.4%)	
Endocrine Therapy, n (%)	Yes	1122(75.3%)	73 (76.0%)	0.871
	No	368(24.7%)	23 (24.0%)	
Radiotherapy, n (%)	Yes	459 (30.8%)	37 (38.5%)	0.113
	No	1031 (69.2%)	59 (61.5%)	
Chemotherapy cycle, n (%)	4 cycles	165 (11.1%)	11 (11.5%)	0.988
	6cycles	476 (31.9%)	31 (32.3%)	
	8cycles	849 (57.0%)	54 (56.3%)	
Complicating disease, n (%)	0	1195(80.2%)	73 (76.0%)	0.533^a^
	1	252 (16.9%)	21 (21.9%)	
	2	40 (2.7%)	2 (2.1%)	
	3	3 (0.2%)	0 (0%)	
Location of catheter tip, n (%)	Upper part of SVC	78 (5.2%)	8 (8.3%)	0.194
	Middle-lower part of SVC	1412 (94.8%)	88 (91.7%)	
Insertion site, n (%)	Left internal jugular vein	664 (44.6%)	57 (59.4%)	0.005*
	Right internal jugular vein	826 (55.4%)	39 (40.6%)	
Catheter size, n (%)	8-Fr	524 (35.2%)	61 (63.5%)	< 0.001*
	6.6-Fr	780 (52.3%)	33 (34.4%)	
	6-Fr	186 (12.5%)	2 (2.1%)	
Material of the catheter, n (%)	Silicone catheter	524 (35.2%)	61 (63.5%)	< 0.001*
	Polyurethane catheter	966 (64.8%)	35 (36.5%)	
Maintenance interval, n (%)	Every 1 month	475 (31.9%)	31 (32.3%)	0.065
	Every 2 month	463 (31.1%)	39 (40.6%)	
	Every 3 month	126 (8.5%)	3 (3.1%)	
	Every 4 month	167(11.2%)	5 (5.2%)	
	Irregular interval	259 (17.4%)	18 (18.8%)	
Indwelling time, n (%)	≤ 1 year	162 (10.9%)	18 (18.8%)	0.010*
	1–2 years	821 (55.1%)	57 (59.4%)	
	> 2 years	507 (34.0%)	21 (21.9%)	

Complicating diseases included hypertension, heart disease, diabetes mellitus. No complicating disease was coded as 0; at least one complicating disease was coded as 1; at least two different complicating diseases was coded as 2; and three different complicating diseases was coded as 3. Indwelling time: the duration from TIVAD implantation to removal or observed events. ^a^Fisher’s exact test. ^*^Statistically significant.

### Multivariate analysis for thrombotic occlusion

Univariate analysis showed that insertion site, catheter materials, catheter size and indwelling time were potential predictive risk factors. These factors were entered in a binary logistic regression analysis. The results showed that insertion site (*P* = 0.004), catheter size (*P* < 0.001), and indwelling time (*P* < 0.001) were independent risk factors for TIVAD thrombotic occlusion (Table 2).

**Table 2:** Risk factors associated with TIVAD-related thrombotic occlusion (multivariate logistic. regression analysis).

Variables	OR (95% CI)	*P *value
Insertion site		0.004
Left internal jugular vein	1	
Right internal jugular vein	0.528 (0.343–0.813)	
Catheter size		< 0.001
6F	1	
6.6F	3.849 (0.913–16.225)	
8F	17.017 (4.075–71.060)	
Indwelling time		< 0.001
≤ 1 year	1	
1–2 years	0.593 (0.333–1.056)	
> 2 years	0.184 (0.091–0.371)	

## Discussion

TIVADs have revolutionized the care of patients requiring long-term venous access, and catheter-related thrombosis (CRT) is the most common complication^9^. A prospective cohort study found that the incidence of catheter-related thrombosis (CRT) was 3.8% in cancer patients, and 9.6% of patients may develop thromboembolism^10^. The incidence of CRT in breast cancer patients is 7.9%^11^. CRT can result in chronic venous obstruction and loss of TIVAD function. Thrombotic occlusion can occur secondary to the thrombotic process, and management of thrombotic occlusion is time-consuming and costly for the health care system. Thrombotic occlusion may cause a delay in oncologic treatment and increase morbidity, leading to potentially life-threatening complications. Therefore, identifying the risk factors for thrombotic occlusion is considered clinically important^5^. To the best of our knowledge, this is the first study to investigate the risk factors for TIVAD-related thrombotic occlusion in the off-treatment period. Based on prospectively collected data on 1586 patients with 96 CRT events (6.1%), our study has shown that the insertion site of the catheter, size of the catheter, and indwelling time are significant risk factors for thrombotic occlusion.

The risk factors for thrombotic occlusion are various, and organic factors include endothelial damage, stasis of venous flow, and/or an underlying prothrombotic state^12^. Other factors associated with the development of thrombotic occlusion in cancer patients include the type of central venous catheter, insertion in the subclavian vein, left-sided insertion, longer duration of catheter insertion, catheter-to-vein ratio, professional expertise, and optimal routine care^13,14^. In the current study, left-sided insertion was found to be associated with a higher risk of thrombotic occlusion. For left-sided insertion, the incidence of thrombotic occlusion was 3.6% (57/1586) compared to 2.5% for right-sided insertion (39/1586), which is consistent with previous studies. Left-sided insertion has been associated with a higher risk of CRT, which may be due to anatomical or technical factors^15–17^. When the left catheter is selected, the length and curvature of the catheter in the blood vessel are increased compared with a right catheter, and the increased catheter length means there is more “space” in which a thrombus can form^18^. Moreover, the catheter inserted through the left side needs to navigate the angles between the left brachiocephalic vein and the SVC, which may lead the catheter tip to remain close to the lateral wall of the blood vessels, resulting in mechanical damage to the endothelium of blood vessels and predisposing to thrombosis^9^. Gravity might be another reason, especially for obese patients. An obese patient lacking a side of the breast for a long time will develop slanting of the body, which increases the pulling strength on the catheter and disengages the catheter from the SVC^15^. Therefore, health care teams should attempt to place catheters on the right if possible.

The diameter of the catheter was another risk factor for thrombotic occlusion. The 2021 INS guidelines^2^. point out that smaller diameters are associated with a reduced risk of catheter-related thrombosis and recommend a catheter-to-vessel ratio of less than 45%. The association between catheter diameter and thrombosis has been confirmed by our study and previous studie^2,18–21^. However, reports on the risk of thrombosis caused by two different catheter materials are inconsistent. A previous study reported that thrombotic occlusion occurred significantly more frequently with SI catheters than with PUR catheters (2.79% vs. 1.33%; *P* < 0.001), whereas PUR catheters are prone to catheter-related venous thrombosis (3.17% vs. 0.74%; *P* < 0.001)^23^. A study conducted by Seckold T. revealed that fibrin sheath formation occurred more frequently in SI catheters than in PUR catheters^24^. Other authors^25,26^ reported that using PUR catheters increased catheter-related thrombosis and infections compared with SI catheters; moreover, there seems to be a close association between thrombosis and infectivity, and venous thrombosis may cause catheter occlusion^27,28^. Reasons that explain these differences are unclear. A study conducted by Panetta D^29^ noted that SI catheters are more sensitive to chemical and physical damage, which results in degradation of catheter material. Nevertheless, SI catheters tend to have larger luminal cross-sections with extension of the indwelling time. However, the PUR catheter performs better than the SI catheter in terms of surface uniformity and material stability. The weakness of the material might be the reason that the SI catheter has a higher chance of developing intraluminal thrombosis and a fibre sheath. The stiffness of the materials might be another reason contributing to the different results^23^. Compared to SI catheters, PUR catheters are stiffer, resulting in mechanical irritation of the vessel wall and increasing the risk of thrombophlebitis. This might be the reason why PUR catheters are prone to develop venous thrombosis. The materials of the TIVADs in the study mainly included silicone (SI) or polyurethane (PUR), and thrombotic occlusion occurred with rates of 3.8% (61/1586) in patients with SI catheters and 2.2% (35/1586) in patients with PUR catheters (*P* < 0.001) during the univariate analysis. However, when multivariate analysis was conducted, the material of the catheter was not an independent factor for thrombotic occlusion. That’ might be influenced by the catheter size during multivariate analysis. In this study, all 8F catheters were made of SI material, and 6F and 6.6F were made of PUR. Further study should consider the interaction between materials and diameters of TIVADs. Despite many reports, there is no consensus on which type of material is better or worse overall, but with increasingly long dwell times, degradation of materials may have an even larger impact on catheter maintenance and related complications^30,31^. Therefore, for longer indwelling catheters, PUR catheters are preferred.

The indwelling time of the catheter was another independent risk factor for thrombotic occlusion according to the results of our study. In our study, most of the patients were positive about expressing their preference to retain TIVADs for the purpose of reuse. Given that the catheter is a foreign surface, the longer the dwell times are, the higher the chances of developing thrombotic occlusion. The association between retention time and thrombosis has been confirmed by many studies^32–34^. However, with the increasing retention times, two main questions remain regarding how to maintain the functional status of TIVADs once they are no longer used for treatments. First, how can the best retention time for breast cancer patients with TIVADs be recommended? In our institution, the retention time of TIVADs is recommended according to the probability of reuse. Generally, a 2-year retention time after adjuvant therapy is recommended owing to the higher risk of relapse within this period. However, for metastatic breast cancer patients, a longer retention time is recommended. To the best of our knowledge, no literature has discussed this issue. Therefore, the best retention time of TIVADs for breast cancer patients needs to be further discussed. Second, manufacturers recommended that TIVADs need to be flushed every 4 weeks to keep the catheter functional^35^. Therefore, a long retention time requires more flushing procedures and higher patient compliance to prevent catheter occlusion. According to a literature review^3^, patients show poor compliance with these time-consuming procedures; in our study, the compliance rate was 31.9% (506/1586). In recent years, many studies have been conducted to find a proper maintenance interval for TIVADs^2,5,36–40^. Although these studies did not recommend a consistent flushing interval, they proved that there was no significant correlation between the flushing interval and thrombotic occlusion. Therefore, to fully answer the question of how long TIVADs should be retained, future studies will be necessary.

Nevertheless, the present study has some limitations. First, it was a retrospective study. Second, the study participants were all breast cancer patients at a single research institution. Thus, multicenter, different cancer types, and prospective studies should be conducted to substantiate these findings in the further. Third, two brands of catheters were included in the study; hence, these results may not be transferred to other manufacturers of SI and PUR catheters. Finally, patients with asymptomatic thromboembolism might be possible to be distinguished, further study assessing the asymptomatic catheter-related thrombosis by a regular vascular examination without causing negative effects is essential.

## Conclusions

TIVADs are an integral part of cancer care. Therefore, maintaining the functional status of TIVADs is crucial. In this study, we clarified that risk factors for TIVAD-related thrombotic occlusion were associated with the left-side insertion, larger size of catheter, and longer indwelling time. Those risk factors offer a clinical value in directing personal treatment for breast cancer patients when TIVAD implantation is needed.

## Methods and materials

### Study population

A retrospective observational study was conducted in the Fourth Affiliated Hospital of Hebei Medical University, where TIVADs were predominantly inserted to facilitate the safe administration of chemotherapy agents. A consecutive series of breast cancer patients with TIVADs implanted and maintained in the Fourth Affiliated Hospital of Hebei Medical University during the period of 1 January 2019 to 31 August 2021 were enrolled. The inclusion criteria for this study were as follows: (1) adult breast cancer patients aged ≥ 18 years old; (2) no previous TIVAD-related complications, in case of the effects of other complications and (3) at least two consecutive maintenance intervals during the study period^36^. Patients who had a history of mechanical occlusion or previous thrombosis, were exposed to anticoagulant therapy, or had haematologic malignancies or other types of malignant tumours were excluded from the study. For patients who underwent more than one TIVAD implantation during the study period, the first implantation data were collected and analysed.

All TIVADs were implanted by expert physicians within the breast centre of the Affiliated Hospital of Hebei Medical University using an ultrasound-guided method.

### Data collection

A Microsoft Excel form was used to extra details from all enrolled patients by one reviewer from patients’ medical cases, nurses’ records and TIVADs maintenance manual, and checked by second reviewer. Among variables extracted were patient-related characteristics at baseline and potential risk factors for thrombotic occlusion reported in the literature^2^: patient-related characteristics (age, body mass index (BMI), menopausal status, exposure to endocrine therapy or radiotherapy, hypertension, diabetes mellitus, heart disease, chemotherapy cycles) and catheter-related data (position of the catheter tip, insertion site, size and materials of the catheter, maintenance interval and indwelling time). Patients were classified into the occlusion group or nonocclusion group based on whether thrombotic occlusion was formed.

### Detecting thrombotic occlusion

This research defined thrombotic occlusion by symptoms including redness, heat, pain, and swelling at the catheter site or the absence of blood return with or without infusion difficulties^2^. Thrombotic occlusion was confirmed by angiography^2^.

### TIVAD maintenance

Off-treatment breast cancer patients with TIVADs were scheduled to the outpatient clinic for routine follow-up according to the protocol adopted at our hospital. When visiting our outpatient clinic, all patients were assessed for any signs of complications, such as oedema or redness at the TIVAD site or any signs of TIVAD-related infection. A standard maintenance procedure was performed by qualified oncology nurses following standard sterile precautions, and the maintenance procedure for TIVADs was the same for all participants. A Huber needle was inserted through the skin to the septum after skin disinfection. Blood return was assessed first to clarify the patency of the catheter. The presence of blood return without any difficulties was recorded on the patients’ maintenance manual as a patent catheter, and standard flushing and locking procedures were performed thereafter, including manually flushing the catheter using 10 cc normal saline with a pushing and pausing method, followed by locking the catheter with 2–3 ml heparin saline (100 U/ml). After flushing and locking the catheter, the Huber needle was removed, and a sterile dressing was applied to protect the skin from infection. Patients with signs of absence of blood return with or without infusion difficulties were further referred to angiography to diagnose the catheter status. Thrombotic occlusion was safely treated according to the protocol adopted by our hospital (urokinase 5,000 U/ml) to restore the patency or removal of the TIVAD). All patients were assessed until the study ended or until TIVAD-related complications occurred. The observation period was defined as the time from the study started to the end of the study, or observed events occurred, such as TIVAPs being removed or reused, or thrombosis occlusion occurred.

### Types of TIVADs

The TIVADs used consisted of 8-Fr silicone (SI) catheters and 6-Fr polyurethane (PUR) catheters from Bard Inc., USA, and 6.6-Fr PUR catheters from Pfm Medical Inc., Switzerland.

### Ethical approval and consent to participate

Written informed consent was obtained from all patients analysed in the present study. The study was approved by the Ethics Committee of the Fourth Affiliated Hospital of Hebei Medical University (approval number: 2021KY248). The study was performed in accordance with relevant guidelines and regulations.

### Statistical analysis

Statistical analysis was performed using SPSS 25.0 (SPSS Inc., Chicago, IL). Categorical data are expressed as frequencies and percentages, and the chi-square test and Fisher’s exact test were used to compare the differences between the nonocclusion group and the occlusion group. The logistic test was used to analyse the independent risk factors for thrombotic occlusion. The adjusted odds ratio (OR) is presented with 95% confidence intervals (CI), and *P* < 0.05 was considered statistically significant.

## Data Availability

The datasets used and/or analysed during the current study are available from the corresponding author on reasonable request.
